# Experts’ Misinterpretation of Box Plots – a Dual Processing Approach

**DOI:** 10.5334/pb.az

**Published:** 2014-11-20

**Authors:** Stephanie Lem, Patrick Onghena, Lieven Verschaffel, Wim Van Dooren

**Affiliations:** 1 Centre for Instructional Psychology and Technology, KU Leuven, Belgium; 2 Methodology of Educational Sciences Research Group, KU Leuven, Belgium

**Keywords:** Experts, Box plots, Dual process theories, Heuristic reasoning

## Abstract

Recent studies have shown that students often misinterpret the area of the box in box plots as representing the frequency or proportion of observations in that interval, while it actually represents density. This misinterpretation has been shown to be based on the saliency of this area and can be explained by heuristic reasoning as defined by dual process theories. In this study we tested whether expert users of box plots also display this misinterpretation and show signs of the same heuristic reasoning as found in students. Using a reaction time test, we found signs of heuristic reasoning in experts, both with respect to accuracy and reaction times. If even experts have difficulty interpreting box plots, one can question whether these are an appropriate form of representation to use when reporting data and deserve the prominent place they currently have in the statistics curriculum.

## Introduction

According to Wilkinson and The Task Force on Statistical Inference (1999), box plots should be used more often in research articles: ‘There are other ways to include data or distributions in graphics, including box plots …. It is time for authors to take advantage of them and for editors and reviewers to urge authors to do so’ (p. 607). However, recent studies have shown that box plots are not easy to interpret at all (e.g., Bakker, Biehler & Konold, 2005; Lem, Onghena, Verschaffel & Van Dooren, 2012, 2013a, 2013b). Various misinterpretations of box plots have been reported in high school and university students. For example, students think that the whiskers do not represent any data, or that the middle line represents the mean rather than the median. Additionally, they think that every value between the minimum and maximum has necessarily been observed.

**Figure 1:**
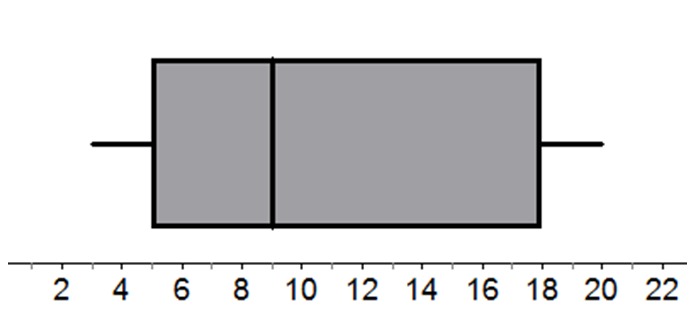
Example of a box plot.

In this study we investigated the occurrence of one specific misinterpretation in expert users of box plots, namely that the area of the box represents the frequency or proportion of observations, while it actually represents their density^1^. Box plots (see Figure 1 for an example) provide a summary of a data set using five data points: the minimum, the first quartile (Q1), the median, the third quartile (Q3) and the maximum. These five figures divide the data set into four intervals containing more or less the same number of observations. Looking at the box plot in Figure 1, the fact that the 9–18 interval is larger than the 5–9 interval indicates a relatively low density in the 9–18 interval. However, various studies have shown that students tend to interpret this larger area as representing more observations than the smaller area (Bakker et al., 2005; Lem et al., 2012, 2013a, 2013b). This means that, in Figure 1, students think that there are more observations above than below the value of nine.

### Heuristic reasoning

The dual process theoretical framework helps to understand why people who in theory possess the required knowledge to solve a certain problem may fail to give a correct answer due to the impact of heuristic processing of certain salient, but not necessarily relevant, problem characteristics. Showing strong relations to Fischbein’s theory of intuitions in mathematical reasoning (Fischbein, 1987), this framework has also recently been applied successfully to mathematics education (e.g., Gillard, Van Dooren, Schaeken & Verschaffel, 2009; Inglis & Simpson, 2004; Leron & Hazzan, 2006; Vamvakoussi, Van Dooren & Verschaffel, 2013). According to dual process theories of reasoning, one tends to use very fast reasoning processes — called heuristic processes — by default when interpreting a situation or task, and only in some cases one will also employ slower and more effortful analytic reasoning processes. Heuristic reasoning processes very frequently lead to the correct solution (the heuristic system probably originates and survives because of its relative effectiveness at high speed with low effort), but analytic reasoning is necessary when heuristic reasoning is not successful. Many variations of dual processing theories of reasoning exist. The main difference between the various theoretical accounts relates to the way heuristic and analytic reasoning are thought to interact. The revised and extended heuristic-analytic model of Evans (2006), which is used as the more specific theoretical framework of the present study, combines many of these theoretical accounts into one model. According to Evans’ model, heuristic and analytic reasoning work in constant competition and interaction with each other. When confronted with a task, one will immediately and in a heuristic mode start to construct the most plausible or relevant *default* model, based on automatically processed salient task features, goal and background knowledge. It is only after this initial heuristic processing that analytic reasoning may also be initiated, depending on many factors such as general intelligence, time available and the instructions that were given for the task. When analytic reasoning takes place, the validity of the default model is evaluated (based on the amount of cognitive conflict experienced) and possibly modified before a final response is given.

An important consequence of Evans’ extended model is that even when analytic thinking takes place, reasoning is still based on — and therefore biased by — the default model and (possibly irrelevant) salient task features. This makes it possible that heuristically processed features still interfere with the analytic stage of the reasoning process and have an important influence on the final outcome of the reasoning. Even experts, who are able to correctly solve the mathematical tasks at hand, could hence still be influenced by intuitions or heuristic reasoning.

A frequently used method to study heuristic reasoning is the comparison of accuracy and reaction times in tests involving two types of items: congruent and incongruent items. For congruent items, the correct test response is the same as the response that would result from the heuristic reasoning that is hypothesised, whereas for incongruent items the correct response cannot be given on the basis of the hypothesised heuristic reasoning alone but requires additional analytic reasoning. This means that one can expect higher accuracy for congruent items than for incongruent items, while reaction times of correct responses to incongruent items can be expected to be relatively long, as more time-consuming analytic reasoning is necessary, compared to congruent items in which fast heuristic reasoning suffices.

**Figure 2:**
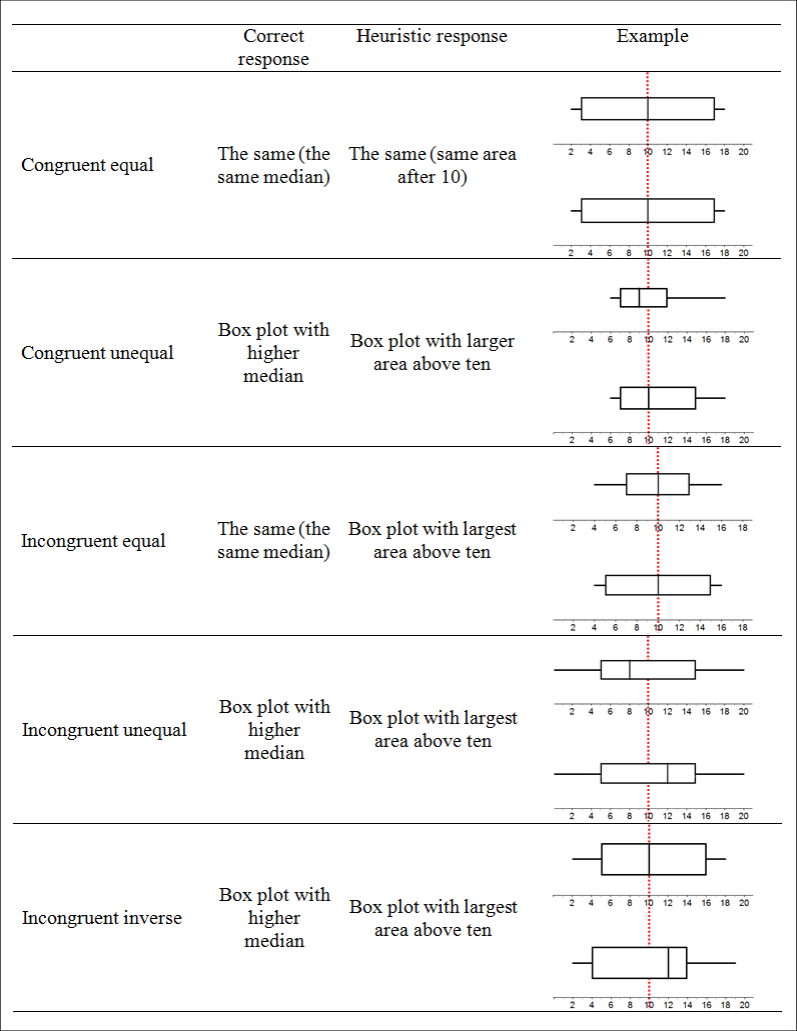
Overview of the five different item types used. The task was to decide, for each pair of box plots representing the exam results of two groups of students, which group had most students with a score above 10.

We recently conducted two experiments that demonstrated the heuristic nature of the area misinterpretation of box plots and its occurrence among students (Lem et al., 2013b). In the first experiment, students were confronted with situations in which they had to compare two box plots (see Figure 2 in the Method section for sample items). Besides accuracy, reaction times were logged. As expected, congruent items were solved significantly better than incongruent items, and correct responses to congruent items were given significantly faster than correct responses to incongruent items, suggesting that slower analytic processing had to take over to correctly solve incongruent items. In the same experiment, another group of students was experimentally stimulated to employ analytic reasoning by receiving a warning about the misleading nature of many graphs and being urged to avoid making mistakes as much as possible. Surprisingly, although reaction times went up in this group, the number of occurrences of the area heuristic – leading to errors on incongruent items – remained at the same level. This suggests that the area heuristic is very difficult to overcome, and all the more strongly so given that all students scored nearly perfectly on a preceding test measuring their factual knowledge about the key elements of box plots. We also manipulated the extent to which the area of the box was salient. In half of the items the area was made less salient by giving this area the same colour as the background colour, while in the other half of the items the area was made more salient by colouring it dark grey. The accuracy and reaction time patterns indicated a stronger inclination to reason heuristically when the area was made more salient. In the second experiment (Lem et al., 2013b), students were involved in an experimental intervention that was aimed at improving their interpretation of box plots and specifically at eradicating the area heuristic. After this intervention, students still showed signs of the area heuristic in their accuracy and reaction time patterns, including those who had scored very high on a preceding box plot interpretation test. This further underpins the hypothesis that the misinterpretation of the box of box plots due to the area heuristic is very persistent. A question that arises next and that will be addressed in the current study is whether it might even still occur in expert users of box plots.

### Graph design principles

In addition to box plots, many other graphical representations have been documented to be misinterpreted by students, for example in mathematics (Acevedo Nistal, Van Dooren, Clarebout, Elen & Verschaffel, 2009; Yerushalmy, 1991) and in biology (Novick & Catley, 2007). Very often, these misinterpretations can be explained by the way people perceive graphical representations (e.g., Elby, 2000). A famous example is given by Bell and Janvier (1981), who report on the use of an item in which a graph representing the speed of a race car as a function of distance is presented together with a question asking how many turns the car has made. Instead of counting the number of times the car has slowed down, participants tended to count the number of ‘turns’ in the graph.

Tversky (1997) argued that such misinterpretations could be avoided if certain principles for designing graphs were respected. She proposed several graph design principles that stem from the idea that the way we interpret graphical representations is based on the way we interpret the world. According to a first principle, both space and direction in graphs should be used in a natural way. Because in real life ‘larger’ usually stands for ‘more’ or ‘better’ (for instance, when making a pile of objects the pile becomes larger with every extra object added), people are likely to interpret a larger area in a graph as representing more observations (while, in box plots, a larger area actually represents a *lower* density of observations).

According to another of Tversky’s graph design principles, graphs should not use more dimensions than the number of variables they represent. Histograms use two dimensions and both dimensions are informative: while one dimension represents the values taken by the observed variable, the other represents the frequency with which these values have been observed. In the case of box plots, however, a two-dimensional box is shown while only the width of this box (and of the associated whiskers) is actually informative. The very salient second dimension of height may give the false impression that two variables are shown and that the height of the box is important too. This principle concerning the restriction of the number of dimensions used in a graph to the number of variables represented has also been proposed by various other authors (e.g., Kosslyn, 2006; Robbins, 2005; Tufte, 1983).

As is clear from the last two sections, the misinterpretation of box plots seems to be based on the saliency of the area of the box and is heuristic in nature. It is for this reason that we have referred to this misinterpretation as the ‘area heuristic’ throughout the present article.

### Focus of the present study

Research on expertise in various domains has revealed that experts focus more on the structural principles of a task, while novices rely more on its surface features (Hardiman, Dufresne & Mestre, 1989; Inglis & Alcock, 2011; Rabinowitz & Hogan, 2002, 2008). An example of this focus on structural principles in the domain of statistics can be found in a study conducted by Rabinowitz and Hogan (2002). University students with varying levels of experience in statistics had to match various statistical problems to each other. While the more experienced students focused on more structural features of the presented problems (e.g., type of test to be used to solve the question), less experienced students tended to focus more on surface features, like the narrative cover story of the problem.

Applying the insights of expert research to the topic of the present study, one would expect that – unlike students – expert users of box plots are no longer hampered by the area heuristic, since it relates to a superficial feature of box plots. Owing to their extensive experience with box plots, experts would be assumed to immediately and automatically look at the correct task feature, i.e., the position of the median, which is also clearly visible in the box plot.

However, if experts are still affected by the area heuristic, they should show the same effects on congruent and incongruent items as the students in our previous experiments (Lem et al., 2013b), albeit perhaps to a lesser extent. Therefore, if experts perform less accurately on incongruent items than on congruent ones, or if their response times on correctly solved incongruent items are longer than on correctly solved congruent items, we have to conclude that the area heuristic continues to play a significant role in experts’ reasoning processes about box plots. The study described in this article was set up to look for such evidence of the occurrence of the area heuristic in experts.

## Method

### Participants

The participants were 40 students and staff of the KU Leuven who could be considered as expert users of box plots. We defined expert users of box plots as people who work with box plots on a regular basis. This was verified in participants, and they also received a box plot knowledge test which asked about some factual information regarding the key elements in box plots, such as the name of these elements (see Lem et al., 2013b). Based on this test, we removed five participants from our analyses, as they scored 50.0% or less. The remaining 35 participants were: students of a master’s of statistics program (*n* = 3), researchers in statistics (*n* = 8), statistics professors (*n* = 9), and researchers in subfields of psychology, sociology and educational sciences where box plots are regularly used (*n* = 15). These participants scored on average 95.4% correct on our box plot factual knowledge test with a minimum of 70.0% (*n* = 2) and a maximum of 100.0% (*n* = 28). Participants were recruited by e-mail and participated on a voluntary basis.

### Materials

The accuracy and reaction time test was exactly the same as the one used in the experiments of Lem et al. (2013b). Participants were given 40 items in which two box plots representing the fictional exam results of two student groups were presented. The participants’ task was to determine which of the two groups had the most students with a mark higher than 10 out of 20. A vertical red dotted line was placed in each box plot item to indicate the score of 10, in order to assist participants in focusing on the comparison of the box plots. Five different item types were constructed: two congruent item types and three incongruent item types. In congruent items the correct response was the same as the heuristic response, while these two responses differed from each other in incongruent items. Examples of the five item types, including their correct and heuristic responses, are provided in Figure 2. The difference between the box plots in different item types is in the position of the first and the third quartiles relative to the median. The area of the box was not manipulated in any other way. In what follows, we explain the different item types and their correct^2^ and heuristic responses.

*Congruent equal* items presented two identical box plots: both the correct and the heuristic responses would be that both box plots have an equal number of students with exam scores above 10. In *congruent unequal* items, the correct response was that one of the box plots represented more students with exam scores above 10 because the median was higher, while heuristic reasoning would lead to the choice of the same box plot because the area of the box at the right of the line marking the 10-score was larger. In *incongruent equal* items the interquartile range of both box plots differed, but the overall range was the same. Because the median was also the same, there was an equal number of exam results above and below 10 in both box plots. The heuristic response here, however, was that one of the box plots had more students with exam scores above 10 because of the larger area of the part of the box situated at the right of the line marking the 10-score in that box plot. In *incongruent unequal* items, both box plots had the same range and interquartile range, but the median was positioned differently. The correct response was that one of the box plots represented more students with exam scores above 10, as the median of that box plot was higher than 10. Heuristically, however, one would think that both box plots represented the same number of exam results above 10, because the area above 10 was the same in both box plots. Finally, *incongruent inverse* items showed two box plots with differently positioned medians and different interquartile ranges. The correct response here was that one of the box plots presented more students with exam scores above 10, while the heuristic response was that the other box plot had more exam results above 10 given the larger area of the box at the right of the line marking the 10-score.

### Procedure

The reaction time test was administered individually at laptop computers in a controlled environment. It started with the presentation of a box plot, naming the elements of the box plot (minimum, Q1, median, Q3, maximum) and reminding participants of the fact that the median represents the middle observation. Next, a general explanation of the task was provided, followed by two sample items for which participants did not receive feedback regarding the correctness of their responses. Finally, the task was summarised and participants were told to work at their own pace and to try to always provide the correct response.

The 40 items were provided in blocks of 10 items each, followed by a break, which participants could end by themselves by tapping the space bar. All items were preceded by a fixation cross which was presented for 500 ms. The items were presented in a semi-random order, with the following restrictions: (a) not more than three consecutive trials with the same item type, (b) not more than three consecutive trials with the same heuristic response, (c) not more than three consecutive trials with the same correct response and (d) not more than three consecutive trials with the same level of congruency. Stickers were placed on keys 9, 6 and 3 of the numerical keyboard, and participants were asked to press key 9 (with sticker reading ‘up’) when their answer was ‘top box plot’, key 6 (with sticker reading ‘=’) when the answer was ‘both the same’, or key 3 (with sticker reading ‘down’) when their answer was ‘lower box plot’. For each item, the participants’ reaction time and accuracy were logged.

### Analysis

Before analysing the data, the reaction times were log-transformed in order to normalise their distribution. Furthermore, all trials with a reaction time more than 2.5 standard deviations from the mean, as calculated within each level of congruency, were considered outliers and were therefore not used in the analyses, resulting in the deletion of 22 (1.57%) trials.

Because our data are clustered, or to state it differently, involve multiple measurements per participant, we opted for multilevel analyses. Multilevel models take into account the possible correlation between the different responses of a single participant (Van den Noortgate & Onghena, 2006). For the analysis of the accuracy rates we used generalized linear mixed models (which can be seen as an extension of logistic regression analysis) with a dichotomous dependent variable (McCulloch & Searle, 2001). For the analysis of the reaction times we resorted to linear mixed models, because of the continuous nature of the dependent variable (Verbeke & Molenberghs, 1997). SAS 9.3 was used for all analyses.

## Results

**Figure 3:**
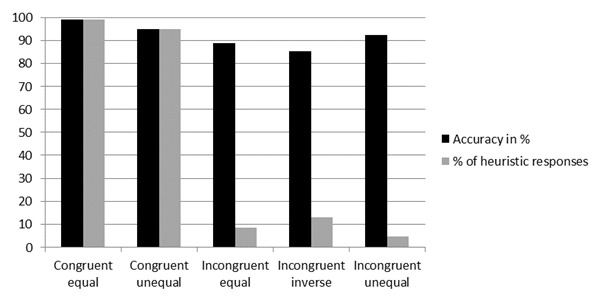
Accuracy rates and number of heuristic responses per item type, in percentages.

Accuracy for the congruent items was 97.8% and it was 95.2% for the incongruent items. A generalized linear mixed model, with accuracy as dependent variable and congruency as independent variable, showed a main effect of congruency on accuracy, *F*(1, 1342) = 7.16, *p* = .008, *OR* = 2.58. This finding indicates that experts are still affected by the area heuristic, although the effect is relatively small, with a difference of only 2.6% between both item types. We also found that 75.0% of all incorrect responses to the incongruent items corresponded to the heuristic response. Both the accuracy rates and the percentages of heuristic responses per item type are displayed in Figure 3.

Using a linear mixed model, the effect of congruency on reaction times for correct responses was analysed. We found a main effect of congruency, with longer reaction times for correct responses to incongruent items (4179 ms, *SD* = 4892.10) than for correct responses to congruent items (3734 ms, *SD* = 3534.01), *F*(1, 1290) = 4.23, *p* = .040, Cohen’s *d* = 0.10. This effect is rather small according to Cohen’s (1988) classification. However, this main effect of congruency on reaction times still indicates that experts need more time to correctly solve incongruent items than to correctly solve congruent items. This effect again points out that experts are affected by the area heuristic, just like novices are, although it also indicates that they are better able to overrule it by analytic reasoning.

## Conclusion and Discussion

Lem et al. (2013b) showed that university students misinterpret box plots due to the heuristic processing of the area of the box, which we termed the area heuristic. In this study we used the same technique to test whether people who can be considered as expert users of box plots still show signs of such an area heuristic. Both the accuracy rates and reaction times in our results show that the area heuristic is not completely eradicated even in expert users of box plots. The accuracy rates revealed that experts sometimes still make the same mistakes as students. The reaction time data indicate that even when correct responses are given, experts first have to overrule the incorrect response previously generated by the area heuristic. It is the demonstration of this last mechanism that is an especially important finding with important implications, as will be discussed below in more detail.

Comparing our results with those of Lem et al.’s (2013b) experiment with novices, we can draw three important conclusions. First, accuracy is notably higher with experts as compared to novices: experts gave 97.8% correct responses for congruent items compared to 90.4% for the novices in Lem et al.’s (2013b) study, and 95.2% correct responses for incongruent items compared to only 63.0% for the novices. Second, a statistically significant difference in accuracy between congruent and incongruent items was still found for the experts just like it was already found for novice users of box plots (Lem et al., 2013b). Although this difference might seem very small and without immediate practical relevance, it still shows the same effect of congruency as with the students and hence provides a first important piece of evidence that the area heuristic is still not totally eradicated in expert users of box plots. The fact that we found this effect even though the experts knew their reasoning was being investigated, most probably making them more cautious, makes it even more plausible that they would also be prone to making the heuristic misinterpretation when less cautious, such as when looking at box plots while reading a research paper. Third, the difference in reaction times for correct responses to congruent and incongruent items was significant in experts in the same way as it was in the novices studied by Lem et al. (2013b). This is a second piece of evidence for the persistence of the area heuristic in experts.

We can conclude that even expert users of box plots are not immune to the fast, incorrect interpretation of the area of the box as representing frequency or proportion of observations. We do see, however, that expert users are better able to overcome this first heuristic interpretation by reasoning analytically. One may argue that in everyday practice, the reaction time difference observed between correct responses to congruent and incongruent items is negligible. Nevertheless, these reaction times indicate that expert users of box plots are still affected by the area heuristic and that, as a consequence, in certain circumstances – for example when under time pressure or when distracted – they may still commit heuristic errors. (This actually occurred in our experiment, where there was a lower accuracy for incongruent items as compared to congruent items). Furthermore, this study showed that this specific heuristic is very persistent; even when one is able to correctly interpret box plots, the incorrect reasoning mechanism still plays a role in the reasoning process. Two important limitations of the current study, however, are the heterogeneous composition of the group of participants and the relatively small sample size. A larger sample size (e.g., more participants in each sub group), would allow for the study of the differences between statisticians and researchers in other subjects, for example. This could be the goal of future research.

The results of this study have important implications, both with respect to the use of box plots in scientific reports and their role in statistics education. With respect to box plots in scientific reports, we can contest the advice of Wilkinson and The Task Force on Statistical Inference (1999) to use box plots in research articles. Our results show that even expert users are to some extent confused by this representation, especially when interpreting box plots swiftly. Moreover, it is very well possible that authors do not use box plots correctly themselves. With respect to the role of box plots in the statistics curriculum, two arguments can be made. On the one hand, it could be argued that if box plots are so problematic even for experts, one might consider removing them from the statistics curriculum in addition to abandoning them from research reports. On the other hand, if the scientific community continues to value and use box plots, it is clear that more time should be given to the instruction of students with regards to this graphical representation. Moreover, research into new methods for teaching box plots, such as the use of multiple representations (e.g., Ainsworth, 2011) or the use of alternative designs of box plots (Tufte, 1983), is necessary as we have shown that the current teaching methods are apparently not successful enough. Some of the many design additions and extensions that have been proposed in the last decades (for an overview, see Wickham & Stryjewski, 2011) could also enable a better interpretation of box plots. However, these plots are usually much more complex to construct and are in some cases hardly recognisable as box plots anymore. Moreover, it should be empirically tested whether these additions and extensions indeed improve interpretation.

## Footnotes

By ‘density’ we mean the relative spread of the data within each interval.

We are aware that, because we chose discrete exam scores (between 0 and 20) of a rather small group of fictional participants as context for our test, it is in principle possible, to construct data sets for the given box plots in some items used in our study that would match any of the answering alternatives by including an extreme number of ties at specific values of the variable under consideration.
